# Osimertinib alone as second-line treatment for brain metastases (BM) control may be more limited than for non-BM in advanced NSCLC patients with an acquired EGFR T790M mutation

**DOI:** 10.1186/s12931-021-01741-9

**Published:** 2021-05-11

**Authors:** Changhui Li, Wei Nie, Jingdong Guo, Anning Xiong, Hua Zhong, Tianqing Chu, Runbo Zhong, Jianlin Xu, Jun Lu, Xiaoxuan Zheng, Bo Zhang, Yinchen Shen, Feng Pan, Baohui Han, Xueyan Zhang

**Affiliations:** 1 Department of Pulmonary, Shanghai Chest Hospital, Shanghai Jiao Tong University, No.241 Huaihai West Road, Xuhui District, Shanghai, 200030 China; 2 Department of Radiation Oncology, Shanghai Chest Hospital, Shanghai Jiao Tong University, No.241 Huaihai West Road, Xuhui District, Shanghai, 200030 China

**Keywords:** Osimertinib, Brain metastases, EGFR T790M mutation, Radiotherapy

## Abstract

**Background:**

This study was designed to investigate the difference between brain metastases (BM) and non-brain metastases (non-BM) treated by osimertinib in advanced patients with an acquired EGFR T790M mutation after obtaining first-generation EGFR-TKI resistance.

**Methods:**

A total number of 135 first-generation EGFR-TKI-resistant patients with an acquired EGFR T790M mutation were retrospectively analyzed. The patients were divided into BM and non-BM groups. According to the type of treatment (whether brain radiotherapy), the BM patients were divided into an osimertinib combined with brain radiotherapy group and an osimertinib without brain radiotherapy group. In addition, according to the type of BM (the sequence between BM and osimertinib), the BM patients were subdivided into an osimertinib after BM group (initial BM developed after obtaining first-generation EGFR-TKI resistance) and an osimertinib before BM group (first-generation EGFR-TKI resistance then osimertinib administration performed; initial BM was not developed until osimertinib resistance). The progression-free survival (PFS) and overall survival (OS) were evaluated. The primary endpoint was OS between BM and no-BM patients. The secondary endpoints were PFS of osimertinib, and OS between brain radiotherapy and non-brain radiotherapy patients.

**Results:**

A total of 135 patients were eligible and the median follow-up time of all patients was 50 months. The patients with BM (n = 54) had inferior OS than those without BM (n = 81) (45 months vs. 55 months, *P* = 0.004). And in BM group, the OS was longer in patients that received osimertinib combined with brain radiotherapy than in those without brain radiotherapy (53 months vs. 40 months, *P* = 0.014). In addition, the PFS was analysed according to whether developed BM after osimertinib resistance. The PFS of the patients that developed BM after acquiring osimertinib resistance was shorter than that without BM development, whether patients developed initial BM after first-generation EGFR-TKI resistance (7 months vs. 13 months, *P* = 0.003), or developed non-BM after first-generation EGFR-TKI resistance (13 months vs. 17 months, *P* < 0.001).

**Conclusions:**

In advanced patients with an acquired EGFR T790M mutation after obtaining first-generation EGFR-TKI resistance, osimertinib may be more limited in its control in BM than in non-BM. Also, osimertinib combined with brain radiotherapy may improve the survival time of BM patients.

## Background

Epidermal growth factor receptor (EGFR)-tyrosine kinase inhibitor (TKI) significantly improves the survival time of advanced non-small cell lung cancer (NSCLC) patients with sensitive EGFR mutation [1, 2]. However, in most patients, the disease progresses after administration of the first-generation of EGFR-TKI for 10–13 months [3]. Due to the presence of a blood–brain barrier (BBB), first-generation EGFR-TKI exerts poor control in the brain [4]. Thus, brain metastasis (BM) is one of the common accounting for 40% of the first-generation EGFR-TKI-resistant metastasis sites [5, 6]. No effective treatment is available once BM has developed, which significantly shortens patient survival time [7]. Therefore, it is critically important to prolong the survival time of BM patients with an acquired EGFR T790M mutation.

The emergence of osimertinib has obviously improved the survival time in BM patients with an EGFR-sensitive mutation and an acquired EGFR T790M mutation [8]. However, due to the high price of osimertinib, it is not cost-effective to use osimertinib as the first-line treatment even in developed countries such as Canada [9]. In developing countries such as China, osimertinib is used mainly for the treatment of patients, especially with BM, with an acquired T790M mutation after obtaining first-generation EGFR-TKI resistance. However, it is still unclear whether osimertinib exerts different effects in advanced BM and non-BM patients with an acquired EGFR T790M mutation after the occurrence of first-generation EGFR-TKI resistance. Furthermore, in the first-generation EGFR-TKI-resistant patients, even if BM did not occur before the administration of osimertinib, it may develop after osimertinib resistance. Nonetheless, it is still not elucidated whether there is a difference in the survival between patients with BM developed before osimertinib administration and those with BM developed after osimertinib resistance.

A previous study showed that first-generation EGFR-TKI combined with radiotherapy (RT) improved significantly more the survival time of BM patients than EGFR-TKI alone [10]. Nevertheless, whether osimertinib combined with other treatments can further prolong survival time is also worth discussing. Further research is required to establish whether osimertinib combined with RT could also bring survival benefits for BM patients with an acquired T790M mutation.

Therefore, this retrospective study was conducted to investigate whether there is a difference between BM and non-BM in the effects of treatment with osimertinib in advanced patients with an acquired EGFR T790M mutation after obtaining first-generation EGFR-TKI resistance. We have also discussed the survival difference between patients with initial BM developed before osimertinib administration and patients with initial BM developed after acquiring osimertinib resistance. In addition, we investigated whether osimertinib combined with brain RT could bring additional survival benefits to the aforementioned BM patients.

## Materials and methods

### Patient selection

From June 1, 2016 to December 31, 2018, a total number of 1180 advanced patients with (EGFR)-mutant lung adenocarcinoma were screened in Shanghai Chest Hospital (Shanghai Chest Hospital, Shanghai Jiao Tong University, PR China). Finally, 135 patients who met the inclusion criteria were enrolled in the study.

The selection criteria were as follows: (1) Patients with stage IV NSCLC; (2) Histological or cytological examinations showed adenocarcinoma, and the gene detection was EGFR-sensitive mutation before initial treatment (19 deletion or 21 L858R mutation); (3) No BM at the initial diagnosis; (4) The first-generation EGFR-TKI was used as first-line treatment; (5) Acquired T790M mutation was detected after obtaining first-generation EGFR-TKI resistance, and osimertinib was applied as a second-line treatment.

The following exclusion criteria were employed: (1) The patients had a resistance mutation or an invalid EGFR mutation; (2) Non-T790M mutation after obtaining first-generation EGFR-TKI resistance; (3) BM developed but not as first-generation EGFR-TKI or osimertinib resistance, or meningeal metastasis developed; (4) Osimertinib was not administered during the treatment or was applied as the first-line treatment; (5) The first-generation EGFR-TKI or osimertinib was combined with other chemotherapy.

The following examinations were used as a clinical baseline assessment for all patients: chest computed tomography (CT), brain magnetic resonance imaging (MRI), bone scan, and abdominal ultrasound. After 4 weeks of treatment with the first-generation EGFR-TKI or osimertinib, the patients were subjected to chest CT and abdominal ultrasound. Then, they underwent chest CT and abdominal ultrasound every 3–4 months in the follow-up treatment until progression occurred. Furthermore, bone scan was conducted every 4–6 months, and brain MRI was done every 3 months. If the patient developed brain symptoms during the treatment, brain MRI was immediately performed to confirm the diagnosis. In addition, the BM patients received MRI every 2–3 months.

### Study design

Patients’ medical records and follow-up data were collected. The following information was recorded: age, sex, smoking history, EGFR mutation type, Eastern Cooperative Oncology Group (ECOG) performance status (PS), time of BM development, BM symptoms, diameter of the largest BM, number of BM, and information on received brain RT or not and RT type. Depending on the BM stage, disease-specific graded prognosis assessment (DS-GPA) was also conducted [11, 12]. The baseline data of patients were classified according to the following criteria: age (< 60 or ≥ 60), sex (male or female), smoking history (yes or no), EGFR mutation (19 deletion or 21 L858R), ECOG-PS (0–1 or 2–3), the largest size of BM (< 1 or ≥ 1 cm), the number of BM (≤ 3 or > 3), BM symptoms(yes or no), DS-GPA score (0–1.5 or 2–4), and information on received brain RT or not and RT type [none, whole-brain radiation therapy (WBRT) or stereotactic radiosurgery (SRS)].

The patients were divided into two groups: patients with BM (n = 54) and patients without BM (n = 81). According to the type of treatment (whether brain radiotherapy), the BM patients were divided into osimertinib combined with brain radiotherapy group (n = 37) and osimertinib without brain radiotherapy group (n = 17). In addition, according to the type of BM (the sequence between BM and osimertinib), the BM patients were subdivided into an osimertinib after BM group (initial BM developed after obtaining first-generation EGFR-TKI resistance, and then taking osimertinib) (n = 33) and an osimertinib before BM group (first-generation EGFR-TKI resistance obtained followed by osimertinib intake and initial BM was not developed until osimertinib resistance had occurred) (n = 21). The study has been approved by the Ethics Committee and the Institutional Review Board, and was carried out in accordance with Helsinki Declaration. An informed consent form was signed by each patient before data collection.

### Evaluation criteria and treatment options

The extracranial lesions were examined and evaluated by chest CT, bone scan, and abdominal ultrasound. The intracranial lesions were assessed by brain MRI. If biopsy could not be performed after obtaining first-generation EGFR-TKI resistance, liquid biopsy (plasma-circulating tumor DNA [ctDNA]) was done. The EGFR mutation detection kit (Amoy Diagnostics, Xiamen, China) was used, based on mutation amplification system technology (ARMS), to detect the most common types of EGFR mutations. The oral doses of the first-generation EGFR-TKI were 150 mg (erlotinib) per day, 250 mg (gefitinib) per day, or 125 mg (icotinib) three times per day. The oral dose of osimertinib was 80 mg per day.

Progression-free survival (PFS) was defined as the time interval between the initiation time of osimertinib administration and the time of osimertinib resistance development. In addition, overall survival (OS) was defined as the time interval from the start of the first-generation EGFR-TKIs application to the death (July 1, 2020). And for patients alive at the data cutoff date, OS was censored at the last follow-up date. The primary endpoint was OS between BM and no-BM patients. The secondary endpoints were PFS of osimertinib, and OS between brain radiotherapy and non-brain radiotherapy patients.

### Data analysis

The categorical variables of patient characteristics were analyzed by χ^2^ test. PFS and OS were assessed by the Kaplan–Meier method, and further comparison was performed using the log rank test. Finally, the Cox proportional hazards regression model was applied for multivariate analysis to determine the independent prognostic factors related to OS. A *P-*value less than 0.05 was considered to indicate a statistically significant difference.

All statistics were analyzed using SPSS software version 23.0 (IBM Corporation, NY, USA), and the Prism software was used for plotting survival curves in this study.

## Results

### Patient characteristics

**Fig. 1:**
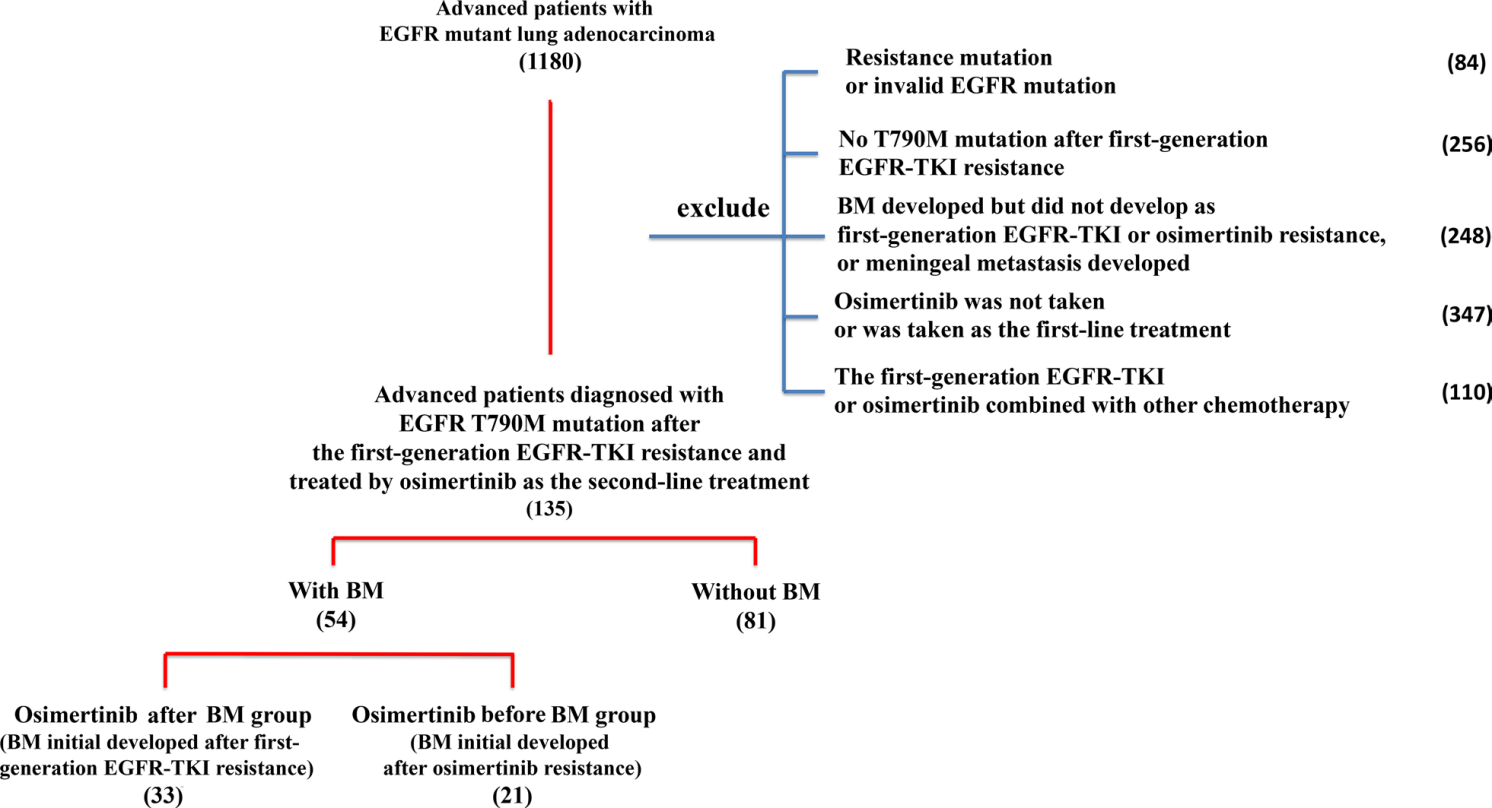
Patients’ selection flowchart

From June 1, 2016 to December 31, 2018, a total number of 1180 advanced patients with EGFR mutation lung adenocarcinoma were screened. Of them, 1045 patients were excluded due to failure to meet the inclusion criteria (Resistance mutation or invalid EGFR mutation (n = 84); No T790M mutation after first-generation EGFR-TKI resistance (n = 256); 248 patients developed meningeal metastasis, or developed BM but did not appear during first-generation EGFR-TKI or osimertinib treatment; 347 patients was not administered osimertinib during the treatment or was applied as the first-line treatment; and 110 patients received the first-generation EGFR-TKI or osimertinib combined with other chemotherapy). Finally, 135 advanced patients, who were EGFR T790M mutation-positive after obtaining first-generation EGFR-TKI resistance and treated by osimertinib as the second-line treatment, met the inclusion criteria and were enrolled for analysis. The flow chart of patient screening is presented in Fig. 1.

Of these 135 patients, 81 (60%) did not develop BM during the entire course of treatment, whereas 54 (40%) developed BM after first-generation EGFR-TKI or osimertinib resistance. Of the BM patients group, 33 patients (24.4%) took osimertinib after initial BM development (BM initial developed after obtaining first-generation EGFR-TKI resistance, followed by osimertinib administration), and 21 (15.6%) developed BM after acquiring osimertinib resistance (first-generation EGFR-TKI resistance occurred, and then osimertinib was administered; initial BM had not been developed until osimertinib resistance had occurred).

The median follow-up time for these 135 patients was 50 months (95% CI 43.45–56.55). The median ages in the BM and non-BM groups were 62 years (36–83 years) and 63 years (35–84 years), respectively. The baseline characteristics of the patients are listed in Table 1. No differences were found in age, sex, smoking history, EGFR mutation and ECOG-PS between the BM group and the non-BM group.

**Table 1:** Patients characteristics

Characteristics	BM group	Non-BM group	P	Total
	No. (%)	No. (%)		No. (%)
Age				
< 60	20 (14.8%)	22 (16.3%)	0.225	42 (31.1%)
≥ 60	34 (25.2%)	59 (43.7%)		93 (68.9%)
Sex				
Male	26 (19.3%)	32 (23.7%)	0.320	58 (43.0%)
Female	28 (20.7%)	49 (36.3%)		77 (57.0%)
Smoking history				
Yes	18 (13.4%)	28 (20.7%)	0.882	46 (34.1%)
No	36 (26.7%)	53 (39.2%)		89 (65.9%)
EGFR mutation				
19 del	30 (22.2%)	53 (39.3%)	0.248	83 (61.5%)
21 L858R	24 (17.8%)	28 (20.7%)		52 (38.5%)
ECOG-PS				
0–1	48 (35.6%)	75 (55.6%)	0.459	123 (91.2%)
2–3	6 (4.4%)	6 (4.4%)		12 (8.8%)
Type of BM^a^				
Osimertinib after BM	33 (61.1%)	–		33 (61.1%)
Osimertinib before BM	21 (38.9%)	–		21 (38.9%)
Size of the largest BM (cm)^a^				
< 1	38 (70.4%)	–		38 (70.4%)
≥ 1	16 (29.6%)	–		16 (29.6%)
Number of BM^a^				
≤ 3	14 (25.9%)	–		14 (25.9%)
> 3	40 (74.1%)	–		40 (74.1%)
Symptom of BM^a^				
Yes	12 (22.2%)	–		12 (22.2%)
No	42 (77.8%)	–		42 (77.8%)
DS-GPA^a^				
0–1.5	15 (27.8%)	–		15 (27.8%)
2–4	39 (72.2%)	–		39 (72.2%)
Radiotherapy (BM)^a^				
WBRT/SRS	37 (68.5%)	–		37 (68.5%)
No	17 (31.5%)	–		17 (31.5%)

*BM* brain metastases, *EGFR* epidermal growth factor receptor, *WBRT* whole brain radiation therapy, *SRS* stereotactic radiosurgery, *ECOG* Eastern cooperative oncology group, *PS* performance status, *DS-GPA* disease specific graded prognostic assessment

^a^Only for patients with BM

### Survival analysis of OS in all patients

**Fig. 2:**
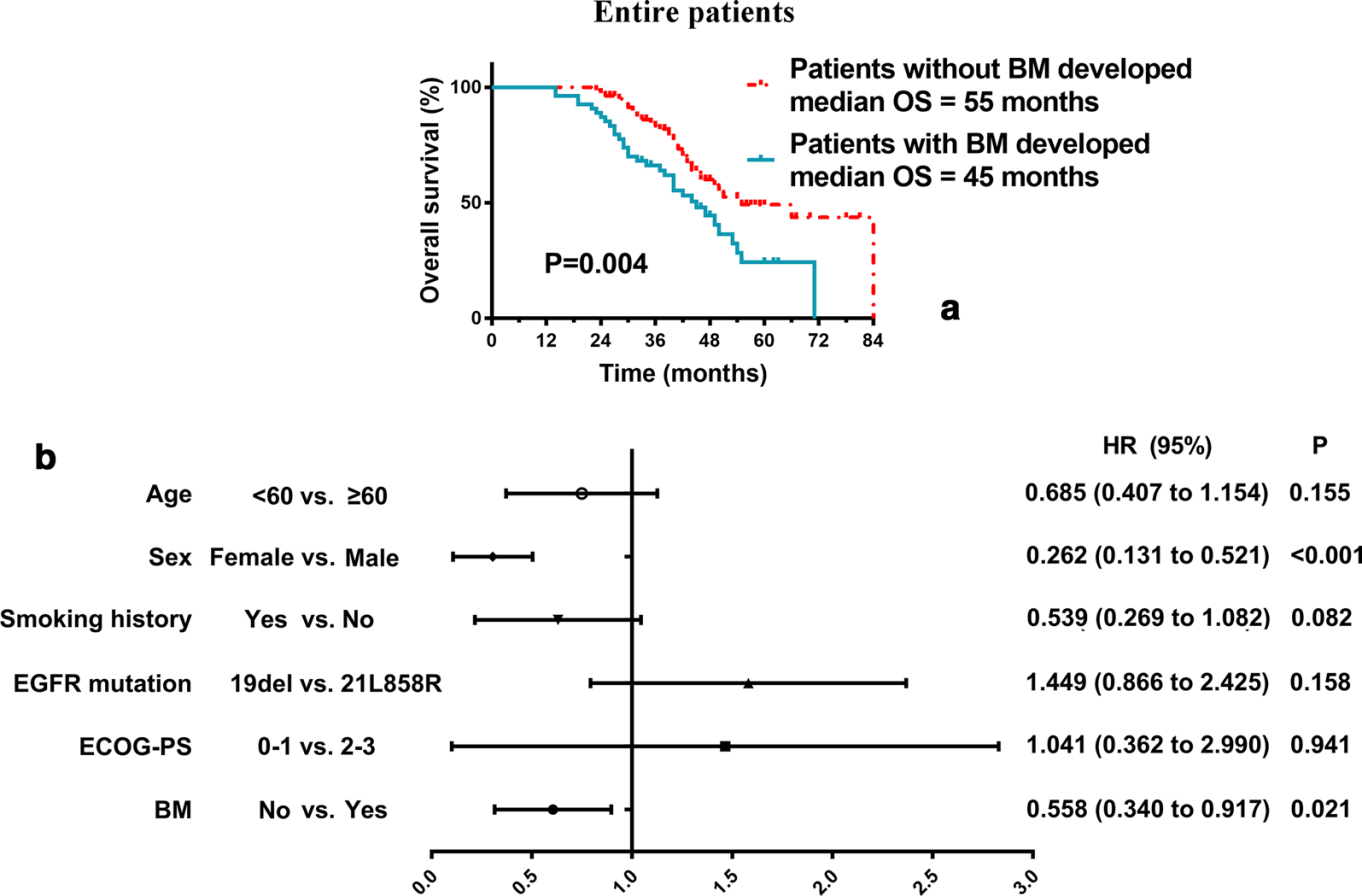
Survival analysis of OS in all patients. **a** The patients without BM had a longer OS than those with BM; **b** In the multivariable model, sex and whether developed BM were independently related to OS among all patients

On July 1, 2020, which was considered the cut-off point, 50% (n = 68) of the patients were still alive (21 cases with BM progression and 47 cases without BM progression). The remaining 67 patients (50%) died during the follow-up period. The median follow-up time in all patients was 50 months; the patients without BM during the treatment had a longer OS than those with BM (55 months vs. 45 months, *P* = 0.004) (Fig. 2a).

After controlling important covariates in the multivariable model, sex and whether developed BM were independently related to OS among all patients (Fig. 2b). Female had significantly longer OS than male (HR 0.262; CI 0.131 to 0.521; P < 0.001). And patients with BM had significantly shorter OS than those without BM (HR 0.558; CI 0.340 to 0.917; P = 0.021).

### Survival analysis of OS in BM patients

The baseline characteristics of the BM patients are presented in Table 2. No differences were established in age, sex, smoking history, ECOG-PS, EGFR mutation, BM-related characteristics, DS-GPA, and BM RT between the osimertinib after BM group and the osimertinib before BM group.

**Table 2:** Characteristics of patients with different types of BM

Characteristics	Osimertinib after BM	Osimertinib before BM	P	Total
	No. (%)	No. (%)		No. (%)
Age				
< 60	12 (22.2%)	8 (14.8%)	0.898	20 (37.0%)
≥ 60	21 (38.9%)	13 (24.1%)		34 (63.0%)
Gender				
Male	18 (33.3%)	8 (14.8%)	0.238	26 (48.1%)
Female	15 (27.8%)	13 (24.1%)		28 (51.9%)
Smoking history				
Yes	13(24.1%)	5 (9.3%)	0.236	18 (33.3%)
No	20 (37.0%)	16 (29.6%)		36 (66.7%)
EGFR mutation				
19 del	16 (29.6%)	14 (25.9%)	0.190	30 (55.6%)
21 L858R	17 (31.5%)	7 (13.0%)		24 (44.4%)
ECOG-PS				
0–1	29 (53.7%)	19 (35.2%)	0.767	48 (88.9%)
2–3	4 (7.4%)	2 (3.7%)		6 (11.1%)
Size of the largest BM (cm)				
< 1	25 (46.3)	13 (24.1)	0.277	38 (70.4%)
≥ 1	8 (14.8)	8 (14.8)		16 (29.6%)
Number of BM				
≤ 3	9 (16.7%)	5 (9.3%)	0.777	14 (26.0%)
> 3	24 (44.4%)	16 (29.6%)		40 (74.0%)
Symptom of BM				
Yes	7 (13.0%)	5 (9.3%)	0.823	12 (22.2%)
No	26 (48.2%)	16 (29.6%)		42 (77.8%)
DS-GPA				
0–1.5	8 (14.8%)	7 (13.0%)	0.467	15 (27.8%)
2–4	25 (46.3%)	14 (25.9%)		39 (72.2%)
Radiotherapy (BM)				
WBRT/SRS	22 (40.7%)	15 (27.8%)	0.713	37 (68.5%)
No	11 (20.4%)	6 (11.1%)		17 (31.5%)

*BM* brain metastases, *EGFR* epidermal growth factor receptor, SRS stereotactic radiosurgery, *WBRT* whole brain radiation therapy, *ECOG* Eastern cooperative oncology group, *PS* performance status, *DS-GPA* disease specific graded prognostic assessment

**Fig. 3:**
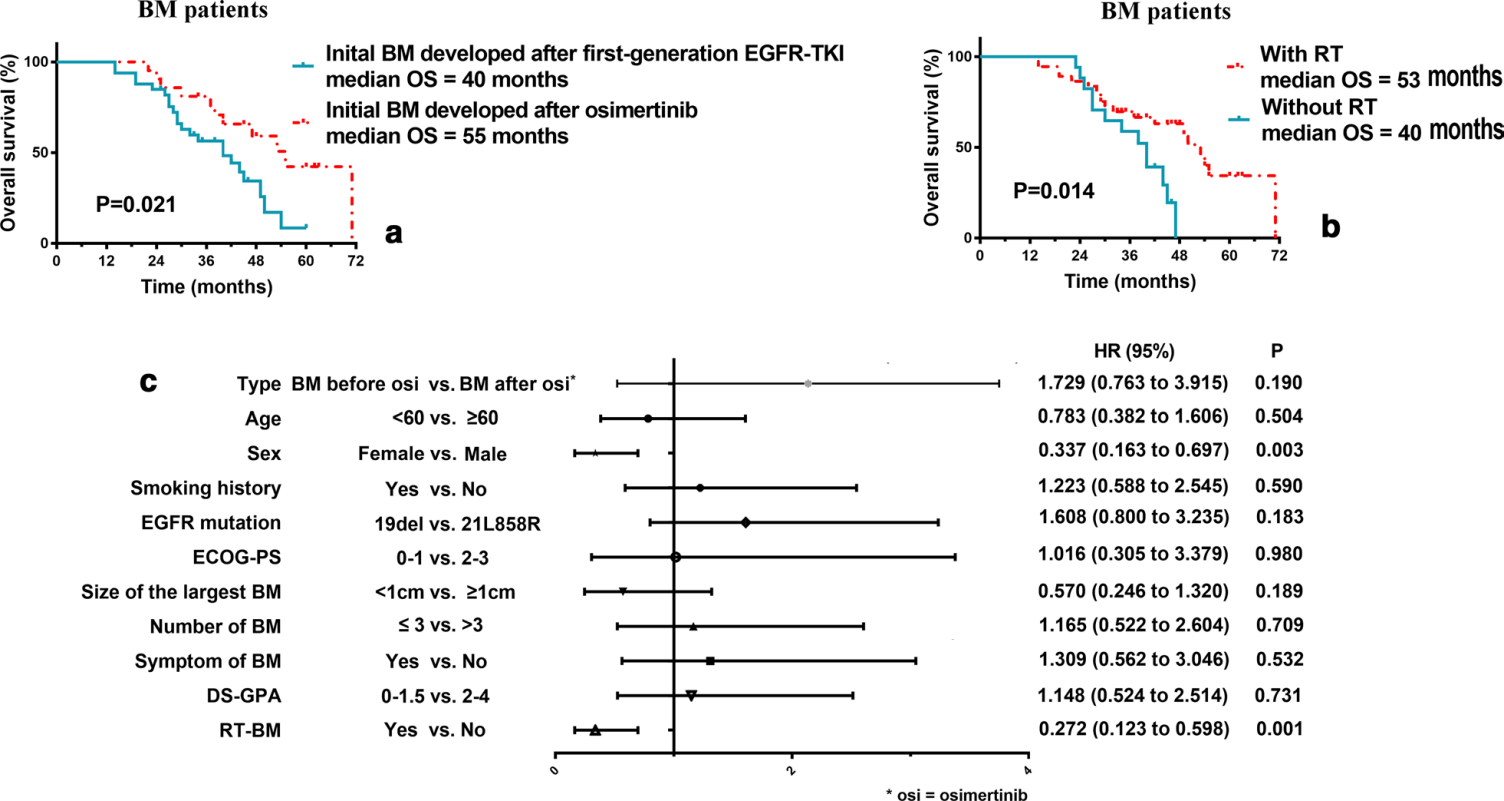
Survival analysis of OS in BM patients. **a** The patients that developed initial BM after first-generation EGFR-TKIs had inferior OS than those that developed initial BM after osimertinib resistance had occurred; **b** The patients receiving RT had a significantly longer OS than those without RT; **c** In the multivariate model, sex, and brain RT were independently associated with OS in the BM patient group

The median follow-up time in the BM patients was 45 months, and the patients that developed initial BM after first-generation EGFR-TKI administration had inferior OS than those that developed initial BM after osimertinib resistance (40 months vs. 55 months, *P* = 0.021) (Fig. 3a). The patients that received brain RT had a significantly longer OS than those without brain RT (53 months vs. 40 months, *P* = 0.014) (Fig. 3b).

Furthermore, in the multivariable model among BM patient group, male was an independent prognostic factor for inferior OS (HR 0.337; CI 0.163 to 0.697; P = 0.003). And brain RT was an independent prognostic factor for superior OS (HR 0.272; CI 0.123 to 0.598; P = 0.001).

### Survival analysis of BM

**Fig. 4:**
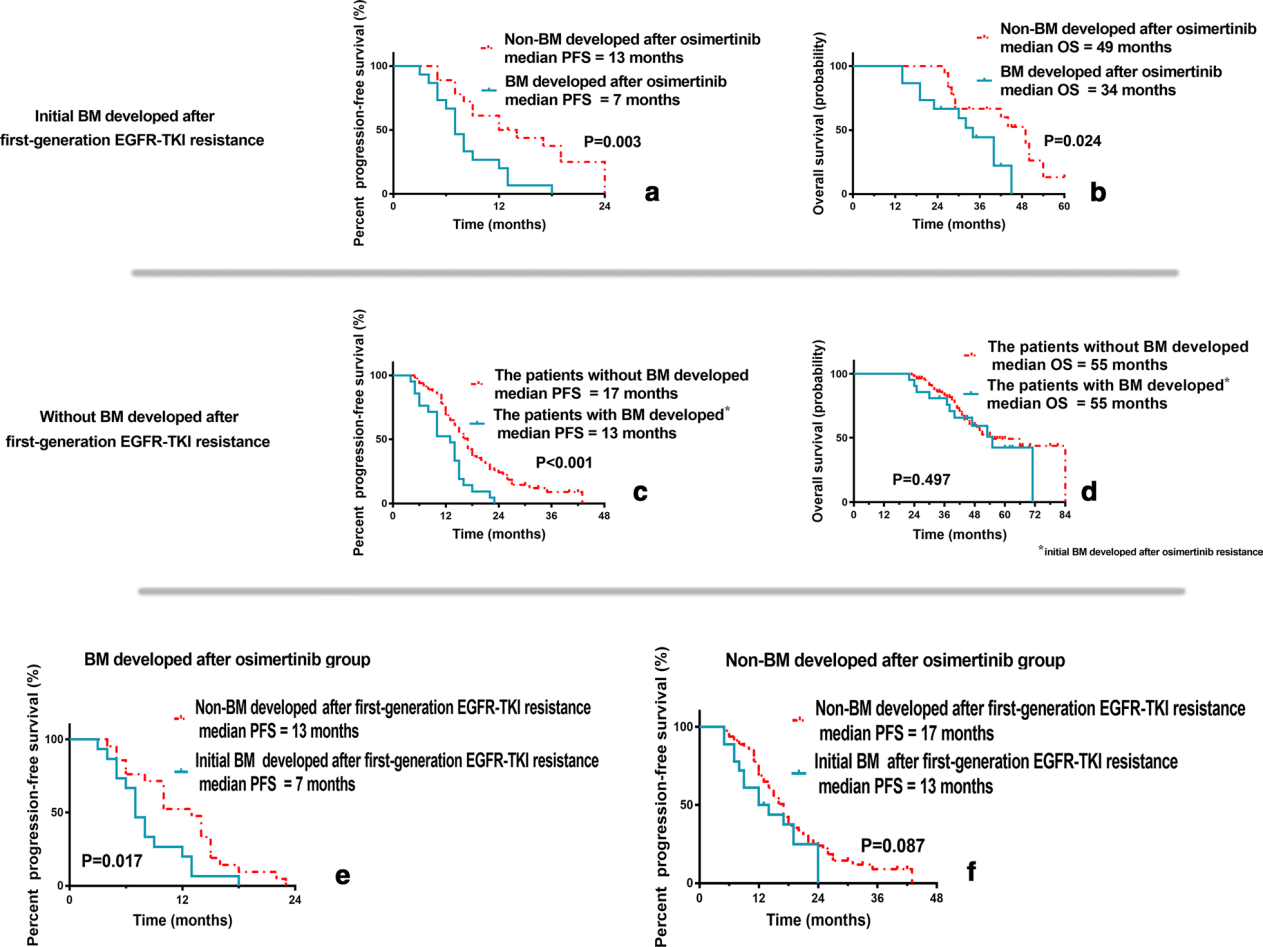
Survival analysis of BM. **a** The patients repeatedly developed BM after acquiring osimertinib resistance had shorter PFS and OS than patients without BM development after acquiring osimertinib resistance; **b** The patients that repeatedly developed BM after acquiring osimertinib resistance had shorter PFS and OS than patients without BM development after acquiring osimertinib resistance; **c** In patients without BM development after obtaining first-generation EGFR-TKI resistance and initial BM developed after acquiring osimertinib resistance, the PFS of the patients with BM developed after acquiring osimertinib resistance was shorter than that without BM development; **d** The patients with initial BM developed after acquiring osimertinib resistance did not show significant OS advantage over the patients without BM development. **e** Of the patients that developed BM after osimertinib group, patients with initial BM developed after obtaining first-generation EGFR-TKI resistance had shorter PFS than those without BM development; **f** In the patients without BM development after osimertinib group, the patients without BM development after obtaining first-generation EGFR-TKI resistance failed to show superior PFS than those with BM developed

The PFS in patients with initial BM developed after obtaining first-generation EGFR-TKI resistance group was 9 months. Of them, the patients that repeatedly developed BM after acquiring osimertinib resistance had shorter PFS and OS than the patients without BM development after acquiring osimertinib resistance (PFS: 7 months vs. 13 months, *P* = 0.003, Fig. 4a; OS: 34 months vs. 49 months, *P* = 0.024, Fig. 4b).

The PFS in patients without BM development after obtaining first-generation EGFR-TKI resistance group was 15 months. And the PFS of patients developed initial BM after acquiring osimertinib resistance was shorter than that without BM development (PFS: 13 months vs. 17 months, *P* < 0.001, Fig. 4c). However, the patients with initial BM developed after acquiring osimertinib resistance did not show significant OS advantage over the patients without BM development (OS: 55 months vs. 55 months, *P* = 0.497, Fig. 4d).

In addition, classification analysis was performed according to whether there was BM developed after osimertinib resistance. In patients developed BM after acquiring osimertinib resistance group, the patients with initial BM after first-generation EGFR-TKI resistance had shorter PFS than those without BM (7 months vs. 13 months, *P* = 0.017) (Fig. 4e). However, in patients developed non-BM after osimertinib resistance group, the patients with initial BM after obtaining first-generation EGFR-TKI resistance failed to show superior PFS than those without BM (13 months vs. 17 months, *P* = 0.087) (Fig. 4f).

## Discussion

In this study, we discuss the efficacy of osimertinib as a second-line treatment in patients, especially BM patients, with EGFR T790M-positive advanced NSCLC who experience disease progression with prior first-generation EGFR-TKI treatment. Finally, 135 patients met the enrollment criteria and were analyzed. Our findings showed that in advanced patients with EGFR T790M mutation after obtaining first-generation EGFR-TKI resistance, osimertinib alone as the second-line treatment to control BM may be more limited in its action than in non-BM patients. The efficacy of osimertinib for patients that had developed initial BM before osimertinib administration was more limited than patients that had developed initial BM after acquiring osimertinib resistance. It is noteworthy that osimertinib combined with brain RT may exert survival benefits in BM patients.

The AURA3 study found that osimertinib had better CNS efficacy in BM patients with an acquired T790M mutation after first-generation of EGFR-TKI resistance, but the treatment of the control group was pemetrexed combined with platinum [8]. The reason for the improved efficacy of osimertinib in BM patients may be the increased concentration of osimertinib in CSF as compared with those of previous drugs (such as first- and second-generation EGFR-TKI or chemotherapy) [13, 14]. However, whether there was a difference in the efficacy of osimertinib as second-line treatment between groups with BM and non-BM lesions is still unclear. Our research revealed that in advanced patients with an acquired EGFR T790M mutation after obtaining first-generation EGFR-TKI resistance, the efficacy of osimertinib as a second-line treatment may be more limited in patients with BM than those without non-BM lesions. In addition, the effectiveness of osimertinib in patients that had developed initial BM before osimertinib administration was more limited than in the patients that had developed initial BM after acquiring osimertinib resistance.

However, the reasons for the difference in the efficacy of osimertinib in patients with BM and non-BM are not yet elucidated. We speculate that the possible reasons might be as follows. First of all, a previous study showed that although the concentration of osimertinib is significantly higher than those of other drugs in CSF, it is still significantly lower than that in the plasma [15]. In addition, the tumor microenvironment of BM lesions may be changed compared with the status of initial lesion, resulting in a poorer effect on BM. For example, unlike in non-BM lesions, BM lesions acquired anti-oxidant gene mutations related to cellular stress response, including Keap-1, Nrf2, and P300, which are key factors for the promotion of secondary metastasis [16, 17].

Since the ability of osimertinib to control BM in patients with advanced EGFR T790M mutations after obtaining first-generation EGFR-TKI resistance may be more limited, than in those with non-BM lesions, a further improvement of the treatment strategy is highly required. First of all, reducing the incidence of BM in patients with a T790M mutation after obtaining first-generation EGFR-TKI resistance is a feasible strategy to improve the survival time. Our previous studies also revealed that first-generation EGFR-TKI combined with chemotherapy as the first-line treatment reduced the incidence of BM and prolonged the time of BM [18, 19]. Furthermore, previous investigations have shown that the combination of RT and first-generation EGFR-TKI have better efficacy than the combination of EGFR-TKI or RT alone [10, 20, 21]. Our previous research also revealed that EGFR-TKI administration after 4 weeks of radiotherapy was more effective [12]. Radiotherapy could not only open the BBB to increase the concentration of intracranial drugs, but also locally control BM [22]. The potential similarity in the effects of osimertinib and RT has not been examined before. In this study, we found that osimertinib combined with radiotherapy in BM patients may lead to better survival benefits than osimertinib alone. However, the time relationship between osimertinib and radiotherapy still needs further exploration in future research. Therefore, for patients without BM, osimertinib alone may provide good survival benefits. But for BM patients, osimertinib combined with RT may be a better treatment option.

Our study also demonstrated that occurrence of initial BM during osimertinib did not affect overall survival for the patients carrying T790M mutation but without BM. Although the underlying mechanism still unclear, we speculated that the difference in treatment time, treatment options (different chemotherapy regimens, osimertinib, radiotherapy, and so on), and treatment sequence after BM progression might affect OS. In addition, the previous studies had established a BM preclinical model based on a multi-organ microfluidic chip, and revealed the potential mechanism of obtaining drug resistance in BM, providing a new strategy for overcoming the treatment resistance of lung cancer BM [23, 24]. Therefore, we could find new effective targets for first generation EGFR-TKI or osimertinib-resistant cells through chip model and select the most effective treatment strategy.

The significance and of our research lies in the fact that relatively novel findings have been reported that can reflect the current status and development of medical practice. However, our research still has some limitations. First, this is a non-random retrospective and single-institution study that includes unrecognized biases and confounding factors. Second, the sample size is relatively small.

## Conclusion

Our research findings reveal that the efficacy of osimertinib alone as the second-line treatment for control of BM in advanced patients with EGFR T790M mutation after obtaining first-generation EGFR-TKI resistance may be more limited than in non-BM cases. The efficacy of osimertinib in the patients that had developed initial BM before osimertinib administration was more limited than in the patients that had developed initial BM after acquiring osimertinib resistance. Importantly, osimertinib combined with brain RT may exert survival benefits in BM patients.

## Data Availability

The enrolled patients agreed to use the information only for this study and refused to share it further.

## Abbreviations

EGFR: Epidermal growth factor receptor
TKI: Tyrosine kinase inhibitor
NSCLC: Non-small cell lung cancer
BBB: Blood–brain barrier
BM: Brain metastasis
RT: Radiotherapy
CT: Computed tomography
MRI: Magnetic resonance imaging
ECOG: Eastern Cooperative Oncology Group
PS: Performance status
DS-GPA: Disease-specific graded prognosis assessment
WBRT: Whole-brain radiation therapy
SRS: Stereotactic radiosurgery
ctDNA: Circulating tumor DNA
ARMS: Amplification system technology
PFS: Progression-free survival
OS: Overall survival
